# Psychometric Validation of the Indonesian Version of the Fear of COVID-19 Scale: Personality Traits Predict the Fear of COVID-19

**DOI:** 10.1007/s11469-021-00593-0

**Published:** 2021-08-23

**Authors:** Nabi Nazari, Shahnaz Safitri, Muhammet Usak, Arman Arabmarkadeh, Mark D. Griffiths

**Affiliations:** 1 Department of Psychology, Faculty of Human Sciences, Lorestan University, Khorramabad, Iran; 2 Department of Educational Psychology, Faculty of Psychology, Universitas Indonesia, West Java, Indonesia; 3 Kazan (Volga region) Federal University, Kazan, Russia; 4 Faculty of Mathematics and Statistics, University of Isfahan, Isfahan, Iran; 5 International Gaming Research Unit, Psychology Division, Nottingham Trent University, Nottingham, UK

**Keywords:** COVID-19, Anxiety, Depression, Fear of COVID-19, Negative affect; Personality, Extraversion, Neuroticism

## Abstract

The coronavirus disease-2019 (COVID-19) pandemic is a global health crisis that has generated fear and negative psychological consequences. The present study evaluated the validity and factor structure of Fear of COVID-19 Scale (FCV-19S) among a sample from the general Indonesian population. The English version of the FCV-19S was translated and back-translated into Indonesian language, followed by a pilot study. Using convenience sampling method, a total of 728 participants completed an online survey distributed on various social media platforms. The survey included the FCV-19S, personality traits (neuroticism and extraversion), Positive and Negative Affect Scale (PANAS), Generalized Anxiety Disorder Scale (GAD-7), and the nine-item Patient Health Questionnaire (PHQ-9). The Indonesian FCV-19S had very good internal consistency (Cronbach’s alpha and McDonald’s omega) and composite reliability (alpha = 0.88, omega = .86, composite reliability = .87). Maximum likelihood confirmatory factor analysis (CFA) was conducted to test construct validity (*χ*^2^/df = 2.51, CFI = .984, SRMR = .028, PCLOSE = .15 > .05, RMSEA = .06, 90% CI [.03, .09]). As for criterion-related validity, the FCV-19S score positively correlated with the score on PHQ-9, GAD-7, negative affect, and neuroticism and negatively correlated with extraversion. Negative affect was identified as the most important predictor of the fear of COVID-19. Personality traits also predicted the fear of COVID-19. The findings provide evidence that the FCV-19S is a reliable and valid instrument for assessing fear generated by COVID-19 among a healthy Indonesian-speaking population.

The coronavirus disease-2019 (COVID-19) has resulted in both a physical and mental health crisis (Wang et al., 2020a). Learning from previous pandemics (e.g., SARS), COVID-19 is much more than just a medical challenge. The potential impact of the pandemic on the mental health of many nations is of increasing global concern (Mamun et al., 2020; Feng et al., 2020). Like any health disaster, the pandemic has generated fear, anxiety, stress, and emotional exhaustion (Satici et al., 2020; Taylor et al., 2020; Arpaci et al., 2020). These negative psychosocial consequences, coupled with uncertainty, can potentially disrupt individual well-being and lead to maladaptive behaviors (Holmes et al., 2020; Li et al., 2020). While health systems around the world have concentrated on treating the physical effects of COVID-19, psychological well-being and emotional aspects also have to be considered (Wang et al., 2020a). Psychological and emotional factors play a substantial role in the success or failure of medical efforts and managing methods of pandemic control (e.g., vaccines and antiviral medication, hygiene practices, and spatial distancing). As a consequence of the pandemic, preliminary psychological research has identified fear as a unique and negative psychological and emotional factor, for all groups and across gender (Martínez-Lorca et al., 2020).

Fear is an automatic adaptive emotional response that occurs in stressful conditions in relation to a specific external danger (Steimer, 2002). In relation to the pandemic, fear can be a motivating factor that facilitates protective and preventive behavior among individuals to avoid infection and in following pandemic health instructions. However, intense and unregulated fear may lead to a series of psychological (e.g., anxiety) and behavioral consequences (Holmes et al., 2020; Sep et al., 2019; Winter et al., 2020; Harper et al., 2020). For instance, excessive fear can undermine complex information processing and social interaction. Fear of infection or infecting family members is one of the most common reactions during pandemics (Ahorsu et al., 2020; Choi et al., 2020) that can result in health anxiety, worry, specific phobias, and psychological distress (Arpaci et al., 2020; Qiu et al., 2020; Fernández et al., 2020; Nazari et al., 2021).

Recently, Ahorsu et al. (2020) developed a brief, psychometrically robust instrument to assess the fear specifically generated by COVID-19 (the Fear of COVID-19 Scale; FCV-19S). The FCV-19S has already been validated in many different countries and across different cohorts with very good psychometric properties (e.g., Martínez-Lorca et al., 2020); Mertens et al., 2020; Soraci et al., 2020; Tzur Bitan et al., 2020). However, there is a lack of information regarding the psychopathological mechanisms (e.g., positive and negative emotion) or personality traits associated with the fear of COVID-19.

Neuroticism and extraversion, in particular, are well recognized as significant predictors of change in positive and negative moods. High negative affect levels (e.g., feeling depressed and feeling ashamed) and mood fluctuations characterize neuroticism. While neuroticism positively predicts depression and anxiety, extraversion negatively affects them. These personality traits can be potentially relevant for understanding maladaptive behaviors during stressful situations (Strickhouser et al., 2017). For example, there is evidence that high neuroticism and low extraversion predict feelings of hopelessness (Chioqueta & Stiles, 2005), which is associated with suicidal behavior (Thakur & Jain, 2020). More specifically, neuroticism has been identified as a vulnerability factor for affective disorders (e.g., depression). Importantly, neuroticism accompanied by unstable negative affective states can elevate anxiety, which is a predictor for suicide (Serafini et al., 2020).

## The Present Study

For the WHO, COVID-19 is much more than a medical challenge. There is also a mental health crisis with possible long-lasting and profound adverse consequences. It is possible that the number of individuals affected by the fear of the pandemic quickly overtakes those infected with it (Grover et al., 2020). Therefore, understanding the pandemic’s effects on psychological health is of great importance (Pfefferbaum & North, 2020; Wang et al., 2020a, 2020b), particularly among vulnerable groups (e.g., adolescents and emerging adults including students) who are generally considered to be a population at risk from a mental health point of view (Brooks et al., 2020).

In early March 2020, the Indonesian government underestimated the dangers of COVID-19, and the virus continued to spread gradually to all Indonesian provinces within a month. At the time of writing (May 2021), Indonesia has the highest number of positive cases among Southeast Asian countries with over 1.42 million cases and one of the world’s highest death rates. The government’s decision, which emphasized economic concerns rather than the health interest of the Indonesian people, contributed to the significant increase of positive cases in Indonesia. At present, Indonesia is in an arguable “do or die” situation because the increasing number of positive cases has almost collapsed the health system. Effective screening is the cornerstone for identifying and then treating pandemic-related psychological and emotional adverse impacts. However, there is a lack of information regarding the psychological impact of the COVID-19 pandemic in the general Indonesian population. Therefore, the present study attempted to validate the Indonesian version of FCV-19S and to evaluate its psychometric properties.

## Method

### Participants

A convenience sample of the general Indonesian population was recruited via social media platforms to participate in the present study. Data were collected from 728 participants (329 males; 399 females) aged 18 to 60 years (*M* = 26.56 years; *SD* = 0.87).

### Measures

Fear of COVID-19 Scale (FCV-19S; Ahorsu et al., 2020) The FCV-19S is a seven-item self-report unidimensional measure that assesses the fear of COVID-19. The scale comprises seven items reflecting emotional fear reactions towards the pandemic (e.g., “*I am afraid of losing my life because of coronavirus-19*”). Items are rated on a 5-point Likert-type scale ranging from 1 (*strongly disagree*) to 5 (*strongly agree*). The total raw scores can range from 7 to 35. Higher scores indicate a greater fear of COVID-19. The psychometric properties of the FCV-19S are reported in “Results.” The Indonesian version of the scale can be found in the Appendix.

The Positive and Negative Affect Schedule (PANAS; Tellegen et al., 1988) The PANAS is a 20-item self-report scale that assesses positive affect (PA; ten items; e.g., *strong*) and negative affect (NA; ten items; e.g., *scared*) and describes different feelings and emotions over the past month. Each item is rated on a five-point scale from 1 (*very slightly*) to 5 (*extremely*). The total raw score on each of the two subscales can range from 10 to 50. Higher scores indicate greater positive or negative experienced feelings in the past month. Internal reliability in the present study was very good (*α* = 0.80).

Brief Patient Health Questionnaire-9 (PHQ-9; Kroenke et al., 2001) The nine-item PHQ-9 was used to assess both major depression and subthreshold depression in the past 2 weeks. Each item (e.g., “*Trouble falling or staying asleep, or sleeping too much*”) is rated on a 4-point scale ranging from 0 (*not at all*) to 3 (*nearly every day*). The PHQ-9 total score ranges from 0 to 27. Higher scores indicate more severe depression symptoms. For the present study, PHQ-9 total scores ≥ 15 were considered as moderately severe to severe depression. Internal reliability in the present study was very good (*α* = 0.85).

Generalized Anxiety Disorder (GAD; Spitzer et al., 2006) The GAD-7 is a brief seven-item scale used to assess the severity of anxiety symptoms over the past 2 weeks. Each item (e.g., “*Worrying too much about different things*”) is scored on a 4-point scale ranging from 0 (*not at all*) to 3 (*nearly every day*). The total scores range from 0 to 21. Higher scores indicate more severe anxiety symptoms. For the present study, GAD-7 total scores ≥ 15 were considered as moderately severe to severe anxiety. Internal reliability in the present study was very good (*α* = 0.83).

Brief 10-Item Big Five Inventory (BFI-10; Rammstedt & John, 2007) The BFI-10 is a brief scale that assesses the “big five” personality traits, two items for each trait. In the present study, only neuroticism and extraversion were assessed. Participants rated two items assessing neuroticism (e.g., “*I see myself as someone who gets nervous easily*”) and two items assessing extraversion (e.g., “*I see myself as someone who has an active imagination*”), on a scale ranging from 1 (*strongly disagree*) to 5 (*strongly agree*). A higher score on each subscale indicates a higher level of that specific individual personality trait. Internal reliability of two dimensions in the present study was very good (*α* = 0.81).

### Procedure

#### Ethics 

All study methods and procedures were reviewed and approved by the research team’s Institutional Human Research Ethics Committee. The committee approved the research protocol to ensure participant confidentiality, sampling, and informed consent. The original scale’s corresponding author granted permission to translate and validate the FCV-19S. All participants provided written informed consent.

#### Transcultural Adaptation of the FCV-19S Scale

Translation of the English version of the FCV-19S was carried out according to international guidelines (Beaton et al., 2000). In the first step, two Indonesian translators independently translated the FCV-19S from English to Indonesian. One of the translators was aware of the concepts being examined in the scale (a psychologist). The other translator was neither aware nor informed of the concepts and had no medical or clinical background. To synthesize a consensus version, an expert committee evaluated both versions. Then, an English translator completed a backward Indonesian-to-English of the consensual version. There were no major changes made during this cultural adaptation.

#### Pilot Study

In the pre-test, a pilot study was performed with 28 participants selected from the target population to test for the readability of the scale to be delivered in an online survey. The participant debriefing was conducted to identify actual and potential linguistic understanding, grammar, and ambiguity.

#### Participant Recruitment 

The study was conducted during the COVID-19 pandemic (July to August 2020), so all data were collected online because face-to-face data collection was not possible. The participants were recruited over a 3-week period. The survey was constructed using *Google Forms* and only distributed online. The recruitment process included advertising the study in online social media (*Instagram*, *Twitter*, and *Facebook*) with links to the survey available on the social media platforms. Also, the link was distributed and posted in online community platforms such as university forums and public forums. Once the link was clicked, it led to an informed consent page to be read and agreed upon by before proceeding to the survey. Only those who provided informed consent given were involved in this study. The informed consent page included information about the study such as the study’s objectives and duration, assurances of anonymity and confidentiality, and voluntary participation. It is also stated that the participants could only complete the survey once. The inclusion criteria were being aged over 18 years, being an Indonesian native, and currently living in Indonesia. A small economic reward was given for completing the survey. A small economic reward was given for completing the survey. The reward was in the form of electronic money in which participants could choose which type of electronic money they used already from the available services in Indonesia. The reward was then transferred to the participant’s electronic money account according to their stated preference.

#### Sample Size 

A priori power analysis for multiple linear regression was calculated using G-Power, using an alpha of 0.05, a power of 0.80, Cohen’s *f*^2^ = 0.02, and four predictors to determine the sample size (Faul et al., 2009). The effect size value (Cohen’s *f*^2^ = 0.02) signifies small effect sizes, according to Cohen’s guidelines (Cohen, 1988). The desired total sample size was 602. In total, 728 participants were recruited in the present study which allowed for a 15% loss of data.

#### Data Analysis

There were no missing values in the assessed variables; therefore, no imputation method was implemented. Descriptive statistics were used to calculate the sample characteristics. Chi-squares, independent *t*-tests, and one-way ANOVAs were carried out to investigate the differences between groups. In the present study, simultaneous total scores of the PHQ ≥ 15 and the GAD-7 ≥ 15 were considered as comorbidity being experienced by the participants. Absolute skewness and kurtosis values assessed the normality assumption. Variance inflation factor (VIF) was utilized to examine the multicollinearity issue (1 < VIF < 3) (Hair et al., 2018). Values of skewness and kurtosis were within <|1| which suggested the absence of severe violations of normality (Tabachnick & Fidell, 2014).

A maximum likelihood confirmatory factor analysis (CFA) was then conducted to evaluate unidimensional construct validity. The following values demonstrate an excellent fitting model (Hu & Bentler, 1999): 1 < *χ*^2^/df < 3, comparative fit index (CFI) > 0.95, root mean square error of approximation (RMSEA) < 0.06, and standardized root mean square residual (SRMR) < 0.06. To evaluate reliability, Cronbach coefficient, composite reliability (CR), and McDonald coefficient (ω) were used. Test–retest reliability was also conducted after 4 weeks. The Cronbach alpha if item deleted values and corrected item correlation values are shown in Table 2. ICC evaluated the test–retest reliability.

To evaluate convergent validity, the average variance extracted (AVE) was calculated (Henseler et al., 2016). To establish criterion-related validity, convergent validity, and discriminant validity of the FCV-19S, correlation analyses were performed using the scores of the GAD-7, PHQ-9, and PANAS. Pearson correlations (*r*) of 0.10, 0.30, and 0.50 correspond to small, medium, and large Cohen’s *d* effect sizes (Cohen, 1992).

A multiple linear stepwise regression analysis was carried out to test the impact of negative affect, personality traits, depression, and anxiety on fear of COVID-19 to investigate the best pattern of variables for predicting fear of COVID-19. The analysis proceeded on a stepwise basis by identifying the best predictors and eliminating the poor predictors. This analysis is particularly appropriate where the objective is to predict with the highest possible accuracy rather than find explanatory models of influences on the dependent variable. All analyses were performed using SPSS version 25 (SPSS Inc., Chicago, IL) and AMOS version 24 with a two-sided 5% level of significance.

## Results

### Descriptive Statistics

The demographic characteristics of the sample are shown in Table 1. The univariate normality of the data was checked. Values of skewness and kurtosis for all 7 items of FCV-19S were within <|1|. The VIF for all 7 items of FCV-19S were in the acceptable range (1 < *VIF* < 3). Significant gender differences were found for anxiety (*t* [726] = 2.63, *p* = 0.01, Cohen’s *d* = *0.3*3, 95% [0.17, 0.49]), negative affect (*t* [726] = 2.75, *p* < 0.01, Cohen’s *d* = *0.3*7, 95% [0.21, 0.53]), and neuroticism (*t* [726] = 2.65, *p* < 0.01, Cohen’s *d* = *0.3*5, 95% [0.19, 0.51]). More specifically, females had significantly higher scores in negative affect (*M* = 31.32, *SD* = 7.51), anxiety (*M* = 9.15, *SD* = 4.88), and neuroticism (*M* = 3.25, *SD* = 0.39) than males’ negative affect (*M* = 29.66, *SD* = 8.01), anxiety (*M* = 8.12, *SD* = 8.01), and neuroticism (*M* = 3.18, *SD* = 0.28), respectively. No other gender differences were found on any other variables. A one-way between-participants ANOVA was carried out to compare the effect of age on GAD-7, PHQ-9, positive and negative affect, and neuroticism. Neuroticism was the only significant difference between age groups (*F* (2, 726) = 8.10, *p* < 0.01). Results also showed that 37% of participants obtained a GAD-7 score ≥ 15 (*n* = 269), and 23% of participants obtained a PHQ score ≥ 15 (*n* = 167). A total of 18.8% participants simultaneously obtained a GAD-7 score ≥ 15 and a PHQ ≥ 15 (*n* = 137; see Table 1).

**Table 1:** Demographic characteristics of the sample (*N* = 728)

Item	Value		Test	*p*-value
Categorical variables				
Gender, n (%)				
Males	243 (45.2)		*χ*^2^ = 80.45	< .001
Females	485 (54.8)			
Age, n (%)				
18 to 24 years	227 (32.6)		*χ*^2^ = 5.15	.07
24 to 30 years	270 (37.0)			
Over 30 years	221 (30.4)			
Education, n (%)				
Primary	240 (33)		*χ*^2^ = 84.48	< .001
Higher education	488 (67)			
Depression, n (%)				
PHQ < 15	561 (77)		*χ*^2^ = 16	< .001
PHQ ≥ 15	167 (23)			
Anxiety, n (%)				
GAD-7 < 15	459 (63)		*χ*^2^ = 11	< .001
GAD-7 ≥ 15	269 (37)			
Comorbidity, n (%)				
PHQ ≥ 15 and GAD-7 ≥ 15	137 (18.8)		*χ*^2^ = 28.97	< .001
Male	37 (30.7)			
Female	100 (69.3)			
Continuous variables *M* (*SD*)				
Age (years)	26.67	5.09	*t*(1, 726) = 1.29	.20
PHQ-9	10.09	5.55	*t*(1, 726) = 1.52	.13
GAD-7	8.81	4.95	*t*(1, 726) = 2.63	.01
FCV-19S	3.11	.41	*t*(1, 726) = 1.80	.07
Neuroticism	3.20	.32	*t*(1, 726) = 2.67	.01
Extraversion	3.44	.71	*t*(1, 726) = .33	.73
PANAS-NA	35.87	6.11	*t*(1, 726) = 2.75	< .01
PANAS-PA	30.77	7.71	*t*(1, 726) = .78	.44

*n*, frequency; *M*, mean; *SD*, standard deviation; *t*, independent *t*-test to compare gender; *FCV-19S*, Fear of COVID-19 Scale, *GAD-7*, Generalized Anxiety Disorder scale; *PHQ-9*, Patient Health Questionnaire; *PANAS-NA*, Positive and Negative Affect Scale; *NA*, negative affect; *PA*, positive affect

### Psychometric Evolution

**Fig. 1:**
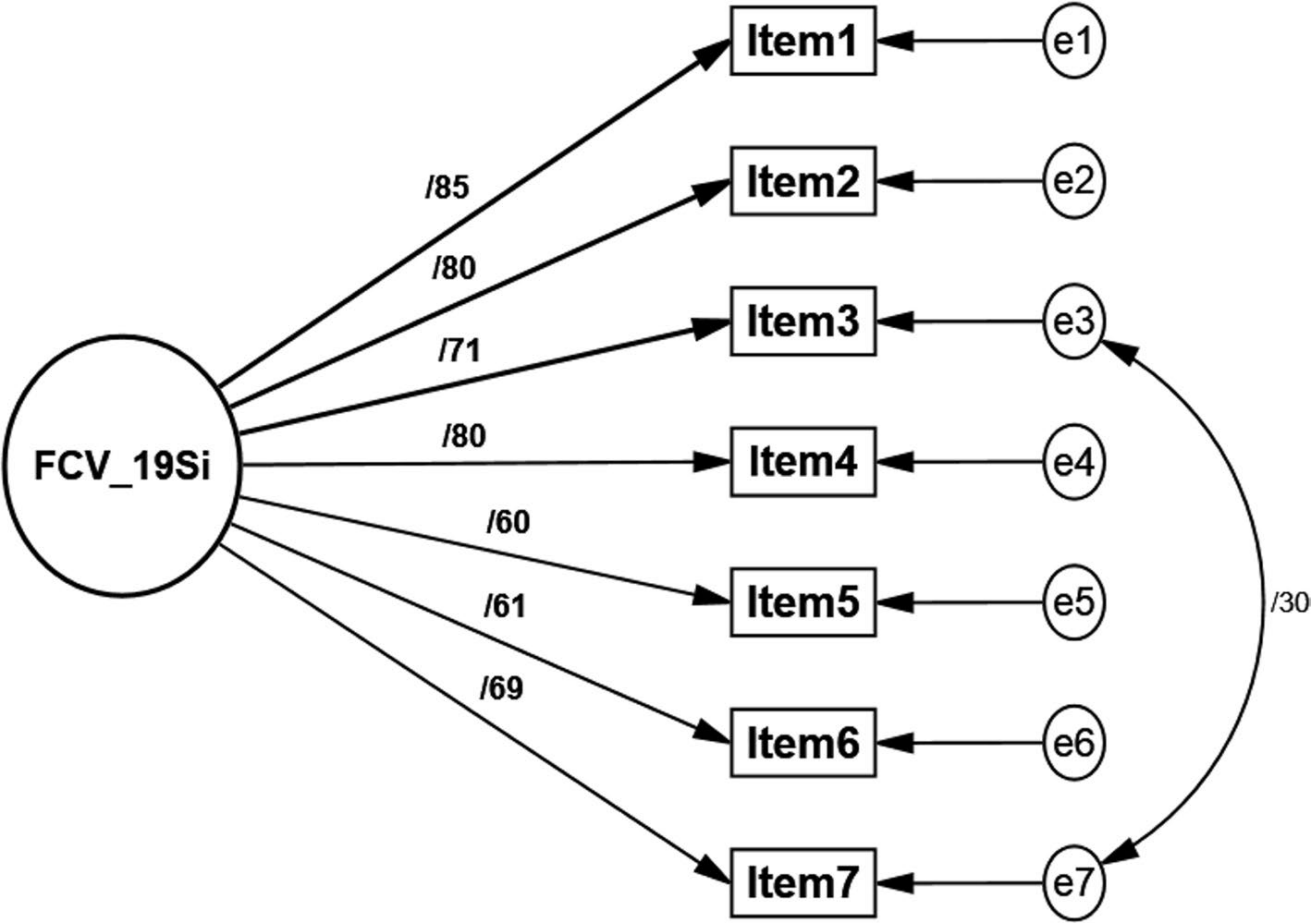
The unidimensional Fear of COVID-19 Scale construct and factor loading

Maximum likelihood (*ML*) CFA on the FCV-19S was robust according to generated values (skewness < 2 and kurtosis < 7) in the normality test (Finney & DiStefano, 2013). The CFA produced an excellent fit for the model in the total sample (*χ*^2^/df = 2.51, CFI = 0.984, SRMR = 0.028, PCLOSE = 0.15 > 0.05, RMSEA = 0.06, 90% CI [0.03, 0.09]). The unidimensional fear of COVID-19 construct was confirmed and is shown in Fig. 1.

The Cronbach alpha of the scale indicated good internal reliability (*α* = 0.88). The Cronbach alpha if item deleted values and the corrected item-total correlations were all well above the recommended level of 0.3 (see Table 2). The intraclass coefficient (ICC) was generated to evaluate the test–retest. After 4 weeks of the original validation study, the survey was sent to half of the participants (*n* = 350) who were randomly selected by a random number generator. Of these, 145 usable questionnaires were returned. In terms of consistency, the ICC was 0.68 with 95% CI [0.59, 0.71] for the single measure. The composite reliability and the McDonald coefficient values were both very good (CR = 0.87, omega = 0.86). The FCV-19S convergent validity was also acceptable (AVE = 0.51).

**Table 2:** Item descriptive statistics

Item number	Item	*M*	*SD*	Skewness^a^	Kurtosis^a^	Corrected item-total correlation	Cronbach’s alpha if item deleted	VIF
1	FCV1	2.31	.98	.20	− .90	.55	.870	1.61
2	FCV2	2.03	1.02	.60	− .67	.56	.870	1.64
3	FCV3	2.06	1.10	.62	− .71	.60	.867	1.89
4	FCV4	2.28	1.03	.34	− .76	.49	.873	1.53
5	FCV5	2.46	.98	.24	− .73	.62	.867	1.85
6	FCV6	2.54	1.05	.03	− 1.02	.53	.871	1.6
**7**	FCV7	2.44	1.03	.19	− 1.05	.46	.874	1.55

*M*, mean; *SD*, standard deviation; *a*, absolute values;

*VIF*, variance inflation factor

As for criterion-related validity, the FCV-19S score positively correlated with the score on PHQ-9 (*r* = 0.24, 95% CI [0.15, 0.22], *p* < 0.01), GAD-7 (*r* = 0.35, 95% CI [0.25, 0.41], *p* < 0.01), neuroticism (*r* = 0.35, 95% CI [0.29, 0.41]**,** *p* < 0.01), and negative affect (*r* = 0.56, 95% CI [0.69, 0.76], *p* < 0.01) and negatively correlated with extraversion (*r* =  − 0.16, 95% CI [− 0.25, − 0.11], *p* < 0.01) and positive affect (*r* =  − 0.05, *p* = 0.56). The full correlation matrix between all the variables is presented in Table 3.

**Table 3:** Correlation matrix of main variables

Measure	FCV-19S	Gender	GAD-7	PHQ-9	PANAS-NA	Neuroticism	Extraversion
FCV-19S	1.00						
Gender	.07						
GAD-7	.37**	.09**					
PHQ-9	.24**	.06	.72**				
PANAS-NA	.53**	.10**	.64**	.52**			
Neuroticism	.35**	.15**	.11**	.06	.13**		
Extraversion	− .16	− .05	− .13	− .15	− .14*	− .09*	
PANAS-PA	− .05	− .03	− .29**	− .37**	− .21**	− .08*	.22**

*FCV-19S*, Fear of COVID-19 Scale; *GAD-7*, Generalized Anxiety Disorder scale; *PHQ-9*, Patient Health Questionnaire; *PANAS-NA*, Positive and Negative Affect Scale (negative affect); *PANAS-PA*, Positive and Negative Affect Scale (positive affect)

^**^Correlation significant at the *p* < .01 level (two-tailed)

^*^Correlation significant at the *p* < .05 level (two-tailed)

The data were subjected to stepwise multiple regression analysis to ascertain the best predictors of Fear of COVID-19. A three-variable model was identified in which negative affect was found to have a *b weight* = 0.03, neuroticism a *b* weight of 0.37, and extraversion a *b weight* = 0.03. Negative affect was entered first and explained 28% of the variance in the fear of COVID-19 [*F*(1, 726) = 281.9, *p* < 0.001; with *R*^2^ change = 0.28, standard error = 0.35]. Second, neuroticism was entered and explained a further 12% [*F* (1, 725) = 93.71, *p* < 0.001]. Finally, extraversion was entered and explained a further 1% [*F* (1, 724) = 9.21, *p* = 002]. In total, the three-variable model explained approximately 37% of the variance in fear of COVID-19 (adjusted *R*^2^ = 0.368). The stepwise regression model coefficients are shown in Table 4.

**Table 4:** Multiple linear stepwise regression analysis (*N* = 728)

Model	Predictor variable	*b*	*SE*	*Beta*	*t*	*p*	*R*	*R*^2^	ANOVA	
									F	df
1	Negative affect	.03	.01	.53	16.79	< .001	.53^a^	.279	281.9	(1,726)
2	Negative affect	.03	.01	.49	16.45	< .001	.60^a^	.360	205.8	(2,725)
	Neuroticism	.37	.04	.29	9.68	< .001				
3	Negative affect	.03	.01	.48	16.08	< .001	.61^a^	.368	141.8	(3,724)
	Neuroticism	.37	.04	.29	9.78	< .001				
	Extraversion	-.05	.02	-.1	-3.03	.002				

^a^Dependent variable: fear of COVID-19

^b^Unstandardized coefficients, *SE*, standard error; *β*, standardized coefficients

*CI*, confidence interval;

*VIF*, variance inflation factor

## Discussion

The optimal dimensions of psychosocial well-being for individuals during the pandemic need empirical investigation. The fear of COVID-19 is associated with an array of negative psychological symptoms, social interference, and physical disability that can compromise psychological well-being. Therefore, the present study was conducted to evaluate the validity and factor structure of the Indonesian FCV-19S. The results demonstrated that FCV-19S is a valid and reliable instrument to assess fear of COVID-19 among the general Indonesian population and confirmed the unidimensional factor structure found in many previous studies in other communities such as Iranian (Ahorsu et al., 2020), Italian (Soraci et al., 2020), Bangla (Sakib et al., 2020), Arabic (Alyami et al., 2020), English (Winter et al., 2020), Turkish (Haktanir et al., 2020; Satici et al., 2020), French (Mailliez et al., 2021), Taiwan (Chang et al., 2020), Malay (Pang et al., 2020), North American (Perz et al., 2020), Cuban (Broche-Pérez et al., 2020), Pakistani (Mahmood et al., 2020), Mexico (García-Reyna et al., 2020), Japanese (Masuyama et al., 2020), and Greek (Nikopoulou et al., 2020; Tsipropoulou et al., 2020). The evaluation of reliability and internal consistency, tested by Cronbach alpha, composite reliability, and McDonald’s construct reliability, all demonstrated that the Indonesian FCV-19S had very good reliability. The Cronbach for the present study (*α* = 0.88) higher than original Persian study (*α* = 0.82, *N* = 771), the Bangla version (*α* = 0.87. *N* = 8550), the Greek version (*α* = 0.87. *N* = 2970), French version (*α* = 0.87, *N* = 371), Turkish (*α* = 0.86, *N* = 668), Italian (*α* = 0.871, *N* = 251), and similar to the Arabic (*α* = 0.88. *N* = 693), and lower than English version (*α* = 0.89, *N* = 1397) (Winter et al, 2020).

To evaluate criterion-related validity, convergent validity, and discriminant validity, the FCV-19S was correlated against measures for depression (PHQ-9), anxiety (GAD-7), negative affect (PANAS-NA), positive affect (PANAS-PA), neuroticism, and extraversion and (as expected) positive associations were found with all of them except positive affect and extraversion. Overall, these findings indicate acceptable criterion-related validity, convergent validity, and discriminant validity.

Significant gender differences were found in total negative affect, anxiety, and neuroticism. These findings concur with previous studies indicating that females report greater psychological problems and are more likely to develop anxiety symptoms and be neurotic compared to males (Blüml et al., 2013). In line with recent studies, females reported more psychological problems associated with COVID-19 than males (e.g., Badahdah et al., 2020; Qiu et al., 2020; Fernández et al., 2020).

Based on the findings, the estimated prevalence of moderately severe to severe depression was 23%, and the estimated prevalence of moderately severe to severe anxiety was 37%. The study’s prevalence was slightly higher than the rates during the COVID-19 pandemic reported among populations in China (Wang et al., 2020a, 2020b; 29% anxiety and 16% depression), Hong Kong (Choi et al., 2020; 14% anxiety and 19% depression), Bangladesh (Islam et al., 2020; 15% anxiety and 18% depression), and Jordan (Naser et al., 2020; 24% anxiety and 13% depression). In addition to the high prevalence of specific mental health disorders, 18.8% of the participants reported moderately severe comorbidity (i.e., they experienced both anxiety and depression).

### Practical Implications

The patterns in relation to psychological reactions to pandemics are complex, and evidence-based treatments must address such complexity. In the present study, greater fear of COVID-19 was associated with a greater negative affect and neuroticism. Neuroticism is a key trait associated with most emotional disorders (Brown & Barlow, 2009; Norton & Paulus, 2017). Other associated factors include anxiety sensitivity, worry, and emotional dysregulation (MacNamara et al., 2015), and have frequently been reported during the COVID-19 pandemic (e.g., Mertens et al., 2020; Restubog et al., 2020; Wu et al., 2021). These factors can be important in increasing or maintaining persistent negative emotions and may affect physical and psychological functioning (Sauer-Zavala et al., 2020). Neuroticism has contributed to the psychopathology of adults during the COVID-19 pandemic and has been a predictor of both depression and generalized anxiety (Nikčević et al., 2021). Individuals high in the trait are sensitive to stress and threats of infection. Consequently, neuroticism may be associated with vulnerability to elevated psychological distress during pandemics. For example, individuals with higher levels of neuroticism and negative affect may be more likely to misinterpret the slightest of non-normal bodily sensations as indications of serious disease (Ferguson, 2009). Therefore, it is not surprising that the severity of neuroticism and negative affects predict their fear of COVID-19.

Anxious or depressive mood states (Barlow et al., 2014) and fears of death and dying in both self and others (Loo, 1984; Zeigler-Hill, & Shackelford, 2020) have been found to be associated with higher neuroticism levels and negative affect in previous studies. The lifetime comorbidity of anxiety disorders with the major depressive disorder may be as high as 73% (Kessler et al., 2005). Given the high prevalence of anxiety and depressive disorders and comorbidities during the current pandemic, disorder-specific interventions may be difficult to justify when the clinical reality is complex, and comorbidities are the norm (Holmes et al., 2018). Transdiagnostic intervention can be potentially served as a promising intervention for individuals psychologically affected by the pandemic. Extraversion traits may facilitate recovery from adverse effects of stressful situations (Nazari & Griffiths, 2020). Individuals with high extraversion traits appear to have a greater tendency to undertake healthy behaviors (Fredrickson & Joiner, 2018) and may have a higher adherence to follow health instructions.

### Limitations

The findings of the study must be interpreted in light of several limitations. The data collected were during the initial COVID-19 outbreak; to minimize infection risk, the data collection occurred online. Using an online data collection method may limit specific relevant population groups (e.g., disadvantaged groups) and other vulnerable groups. Therefore, the data cannot represent the views of these disadvantaged groups and affects the study findings’ generalizability. However, online data collection tends to provide more honest and truthful responses than those utilizing offline methods (Griffiths, 2010). Another limitation of the present study was that the data relied entirely on self-report measures which have well-established methodological biases.

### Conclusion

The findings of the present study provide evidence that FCV-19S is a reliable and valid instrument to assess fear of COVID-19. The Indonesian FCV-19 can be used in the prevention and clinical research in Indonesia. The fear generated by the COVID-19 is also associated with depression, anxiety, negative affect, and neuroticism. The FCV-19S can empirically contribute to deeper understanding psychological consequences of the pandemic. The present findings highlight the importance of personality traits and in fear generated by the pandemic. The FCV-19S can be utilized by mental health staff to identify the risk groups based on sociodemographic characteristics. The present findings highlight the importance of personality traits in fear generated by the pandemic. In line with these findings, psychologists could develop targeted intervention programs to reduce fear generated by the pandemic. Empirically, studies conducted throughout the COVID-19 pandemic would benefit from including an assessment of COVID-19-related fear as outcome variables.

## Appendix. The Indonesian version of the Fear of COVID-19

Instruksi: Dibawah ini terdapat beberapa pertanyaan mengenai perasaan, pikiran, ataupun perilaku Anda terkait dengan pandemik COVID-19. Bacalah setiap pertanyaan tersebut dengan baik dan berikan tanda centang (√) pada satu dari lima pilihan respons yang paling menggambarkan diri Anda saat ini.

**Table:**

Pernyataan	Sangat Tidak Sesuai	Tidak Sesuai	Netral	Sesuai	Sangat Sesuai
1. Saya takut pada COVID-19	□	□	□	□	□
2. Saya merasa tidak nyaman jika memikirkan tentang COVID-19	□	□	□	□	□
3. Tangan terasa lembab ketika saya memikirkan tentang COVID-19	□	□	□	□	□
4. Saya takut akan kehilangan nyawa karena COVID-19	□	□	□	□	□
5. Saat menonton berita atau mendengar cerita tentang COVID-19 di media sosial, saya menjadi gelisah dan cemas	□	□	□	□	□
6. Saya sulit tidur karena khawatir akan terpapar COVID-19	□	□	□	□	□
7. Jantung saya berdebar saat membayangkan terpapar COVID-19	□	□	□	□	□
